# Cadmium-Induced Cytotoxicity: Effects on Mitochondrial Electron Transport Chain

**DOI:** 10.3389/fcell.2020.604377

**Published:** 2020-11-30

**Authors:** Jacopo Junio Valerio Branca, Alessandra Pacini, Massimo Gulisano, Niccolò Taddei, Claudia Fiorillo, Matteo Becatti

**Affiliations:** ^1^Department of Experimental and Clinical Medicine, Anatomy and Histology Section, University of Firenze, Firenze, Italy; ^2^Department of Experimental and Clinical Biomedical Sciences “Mario Serio”, University of Firenze, Firenze, Italy

**Keywords:** cadmium, cytotoxicity, mitochondria, mitochondrial electron transport chain, mitochondrial complexes

## Abstract

Cadmium (Cd) is a well-known heavy metal and environmental toxicant and pollutant worldwide, being largely present in every kind of item such as plastic (toys), battery, paints, ceramics, contaminated water, air, soil, food, fertilizers, and cigarette smoke. Nowadays, it represents an important research area for the scientific community mainly for its effects on public health. Due to a half-life ranging between 15 and 30 years, Cd owns the ability to accumulate in organs and tissues, exerting deleterious effects. Thus, even at low doses, a Cd prolonged exposure may cause a multiorgan toxicity. Mitochondria are key intracellular targets for Cd-induced cytotoxicity, but the underlying mechanisms are not fully elucidated. The present review is aimed to clarify the effects of Cd on mitochondria and, particularly, on the mitochondrial electron transport chain.

## Introduction

Cadmium (Cd) is a toxic heavy metal without known biological function in humans. It can be easily found in house dust and tobacco smoke (Hogervorst et al., 2007), and for this reason, the airways are considered among the primary routes for the entry of Cd. As recently demonstrated in a human airway tissue model (Xiong et al., 2019), Cd impairs cilia functions and enters the lung, where it can induce apoptosis by reactive oxygen species (ROS) production and by altering the reduced glutathione (GSH)/oxidized glutathione (GSSG) redox balance. Once in the lung alveoli and diffused in the bloodstream, Cd is transported to the whole body and, due to its low rate of excretion, can deeply affect kidney functional integrity (Gonick, 2008). As demonstrated in rats (Patra et al., 1999), Cd accumulation in the proximal tubular epithelium of the nephron directly compromises mitochondrial functions (Gobe and Crane, 2010), increasing mitochondrial permeability and swelling, thus inhibiting respiration. These alterations are responsible for an increase in ROS production, ultimately leading to renal cell apoptosis *via* cytochrome c and caspase pathways (Gobe and Crane, 2010; Mao et al., 2011).

Other organs and cellular compartments such as testes (i.e., Sertoli, Leydig, and germ cells) (Zhu et al., 2020), osteocytes (Uwagie-Ero et al., 2019), cardiomyocytes (Chen et al., 2015), and peripheral and central nervous system (CNS; Wang and Du, 2013) also represent main targets of Cd toxicity. A recent meta-analysis has suggested that increased levels of Cd in human serum might play a pivotal role in neurodegenerative disorder progression (Xu et al., 2018). *In vitro* and *in vivo* studies demonstrated a direct role of Cd in ROS production and endoplasmic reticulum stress (Shukla et al., 1996; Branca et al., 2019a), ultimately leading to the impairment of blood–brain barrier (BBB) integrity. Indeed, once the BBB integrity is compromised, heavy metals and other toxicants can enter the CNS and activate glial cells, causing brain inflammation and neural degeneration (Branca et al., 2020). Once inside the brain parenchyma, Cd can induce mitochondrial dysfunction and subsequent cytochrome c release in neural cells (Branca et al., 2018, 2019b).

## Cadmium and Mitochondria

**FIGURE 1:**
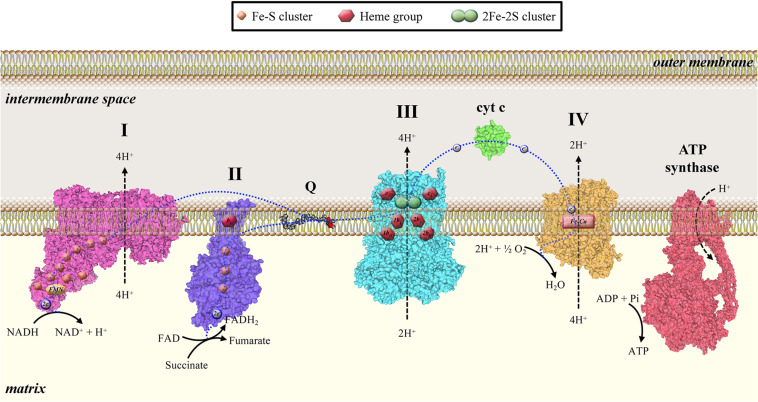
Mitochondrial electron transport chain consists of four complexes: Complex I (NADH-coenzyme Q reductase), Complex II (succinate dehydrogenase), Complex III (coenzyme Q-cytochrome c reductase), and Complex IV (cytochrome c oxidase). Complexes I, III, and IV establish the proton gradient across the IMM for ATP synthesis by ATP synthase.

Mitochondria are organelles of bacterial origin involved in several cellular processes, such as energy homeostasis, cell proliferation, metabolism, and cell death. Mitochondria play a crucial role in the metabolism of eukaryotic cells, producing energy through aerobic respiration. The metabolic energy derived from oxidation of carbohydrates, lipids, and amino acids is used to generate adenosine triphosphate (ATP) through a process called oxidative phosphorylation (OXPHOS) (Milenkovic et al., 2017). This series of oxidation–reduction reactions involves the electron transfer from NADH (nicotinamide adenine dinucleotide) or FADH_2_ (flavin adenine dinucleotide) to molecular oxygen through a series of specific electron carriers that constitute the electron transport chain (ETC)—or respiratory chain—of the inner mitochondrial membrane (IMM). ETC consists of four complexes: Complex I (NADH-coenzyme Q reductase), Complex II (succinate dehydrogenase), Complex III (coenzyme Q-cytochrome c reductase), and Complex IV (cytochrome c oxidase). Complexes I, III, and IV establish the proton gradient across the IMM for ATP synthesis at ATP synthase (Figure 1). Ten protons are transported from the matrix across the IMM for every electron pair transferred from NADH_2_ to O_2_. These protons are utilized by ATP synthase to synthetize 2.5 ATP molecules from ADP and inorganic phosphate (Papa et al., 2006).

Under physiological conditions, the IMM is selectively permeable to solutes and, in mammalian mitochondria, has a cutoff of 1.5 kDa. Apoptotic stimuli, ROS, and mitochondrial Ca^2+^ overload can induce IMM to undergo permeability transition. This sudden increase in the IMM permeability leads to mitochondrial depolarization, inhibition of respiration and ATP synthesis, Ca^2+^ release, osmotic pressure increase, and mitochondrial swelling (Bernardi et al., 2006). Matrix swelling alters the outer mitochondrial membrane (OMM) integrity resulting in apoptotic factor mobilization [cytochrome c (cyt c), apoptosis-inducing factor (AIF), endonuclease G], mitochondrial dysfunction, and cell death (Bernardi and Di Lisa, 2015). The mitochondrial permeability transition is due to opening of the permeability transition pore (PTP), a high conductance channel at contact sites between the IMM and OMM that is crucially regulated by a variety of pathophysiological effectors. However, the molecular structure of the PTP is controversial. It is widely accepted that the voltage-dependent anion channel (VDAC), ATP synthase subunits, and adenine nucleotide translocase (ANT) are components of the PTP complex (Karch et al., 2019).

Mitochondria are among the major sources of endogenous ROS that are formed as natural by-products of physiological ETC activity and participate in cellular signaling (Brand, 2016; Zhang et al., 2016). Reactive oxygen species overproduction can damage cellular lipids, proteins, or DNA, inhibiting their normal function (Valko et al., 2007). Reactive oxygen species exposure has led cells to develop a series of defense mechanisms, called antioxidants (Cadenas, 1997). Under physiological conditions, a balance between ROS production and the intracellular antioxidant levels exists. When this equilibrium is disturbed either by an increase in ROS production or a decrease in antioxidant levels, a condition called oxidative stress, resulting in cell dysfunction or cell death, occurs (Valko et al., 2007).

Belyaeva et al. (2004b) reported that both mitochondrial ETC and membrane permeability are the primary targets of Cd-induced mitochondrial dysfunction. On the other hand, Ciapaite et al. (2009) elegantly demonstrated, in mitochondria isolated from the liver of male Wistar rats, that Cd interferes with mitochondrial respiration solely through the inhibitory effect downstream Q, resulting in respiratory and phosphorylation rate reduction. Therefore, in the presence of a high inorganic phosphate concentration, Cd can induce uncoupling (Koike et al., 1991; Korotkov et al., 1998). However, the effect of Cd on mitochondrial respiration is related to concentration: while a high Cd concentration inhibits basal respiration, a low dose stimulates resting-state respiration (Belyaeva and Korotkov, 2003). Moreover, in the MDA-MB231 human breast cancer cell line, Cd long-term treatment induces an increase in the rate of respiration (Cannino et al., 2008), while short- and long-term Cd treatment in HB2 mammary luminal immortalized epithelial cells decreases respiratory activity and promotes mitochondrial membrane polarization (Cannino et al., 2009).

Many years ago, it was proposed that the toxic effect of Cd could be mainly due to ETC enzyme inhibition (Miccadei and Floridi, 1993) and to proton flux through P_i_/H^+^ symporter leading to an uncoupling effect (Koike et al., 1991). In particular, it has been suggested that Cd, reacting with SH groups from the matrix side of the mitochondrial membrane, leads to Pi/H^+^ symporter conformational change and penetrates into the mitochondria *via* the Ca^2+^ uniporter. This conformational change results in the stimulation of H^+^ and Pi influxes, and the transported H^+^ acts as a trigger for uncoupling (Koike et al., 1991). More recently, new data have highlighted the strict dependence of ETC function on Cd-induced toxicity (Belyaeva et al., 2004a, 2011; Wang et al., 2004; Ivanina et al., 2008; Ciapaite et al., 2009; Adiele et al., 2012; Belyaeva, 2018). Cysteine residues, Fe–S clusters, thiol and binding sites for divalent metals are potential Cd target sites (Kurochkin et al., 2011), leading to ETC inhibition, proton motive force dissipation, and cell dysfunction (Wang et al., 2004; Kurochkin et al., 2011). Moreover, it has been demonstrated that a high respiration rate (expressed in terms of oxygen consumption; nmol O_2_/mg protein/min) promotes Cd accumulation (Belyaeva et al., 2002; Adiele et al., 2010), suggesting that cell energy state influences Cd accumulation. Here, we reviewed the main effects of Cd on mitochondrial ETC complexes.

## Cadmium and Complex I

Mammalian Complex I (NADH-coenzyme Q reductase), with an estimated mass of about 1,000 kDa, is a crucial enzyme in mitochondrial respiration. This giant complex transfers a pair of electrons from NADH to Q to establish the proton motive force across the IMM required for ATP synthesis. Four protons are transported from the matrix across the IMM per two electrons passed from NADH to Q (Sazanov, 2015). It contains 44 polypeptide chains, one molecule of flavin mononucleotide (FMN), and nine Fe-S clusters, together containing a total of 20–26 iron atoms (Agip et al., 2019). On the matrix side of the IMM, the two-electron donor NADH transfers electrons to FMN. Then, the reduced FMN (FMNH_2_) transfers electrons to a series of Fe-S proteins. Fe–S proteins are one-electron transfer agents; therefore, they can transfer one electron at a time to substrate. At this purpose, the flavin of FMN can act as either a one-electron or a two-electron transfer agent linking NADH and Fe–S proteins. Finally, two electrons are transferred from Fe–S cluster to Q.

Cadmium is a powerful uncoupling agent and inhibits the succinate- and malate/pyruvate-stimulated respiration (Müller and Ohnesorge, 1984). Moreover, in rat hepatic mitochondria, it has been suggested that Cd interacts with Complex I at Q site and NADH site levels, resulting in Complex I inhibition (Cameron et al., 1986). The importance of Fe-S clusters for Cd interaction was confirmed by Kurochkin et al. (2011) who demonstrated that the substrate oxidation and proton leak subsystems are the main targets for Cd toxicity in oyster mitochondria. Moreover, Adiele et al. (2012) confirmed the importance of Fe–S clusters for Cd interaction showing, in isolated rainbow trout hepatic mitochondria, the inhibitory effect of Cd on Complexes I, II, and III; the nonresponse of Complex IV could be due to the absence of Fe-S clusters in this ETC complex. On the contrary, no significant inhibition of Complex I activity was observed in Cd-treated mitochondria isolated from the liver, brain, and heart of the guinea pig (Wang et al., 2004). Furthermore, many studies support the involvement of Complex I in mitochondrial PTP and cell death, supporting the direct involvement of the ETC complexes in PTP formation and regulation (Grivennikova et al., 2001; Batandier et al., 2004; Belyaeva et al., 2004a, 2012; García et al., 2005; Belyaeva, 2010). Monteiro et al. (2018) demonstrated, in mitochondria isolated from human osteoblast MG-63 cell line, decreased Complex I and IV activities, leading to an impairment of mitochondria ATP production as a result of Cd treatment. Recently, it was demonstrated that Cd treatment induces ROS production at Complex I flavin site and inhibits respiration in rainbow trout liver mitochondria, confirming the key role of Complex I in Cd-induced ROS production (Okoye et al., 2019).

## Cadmium and Complex II

Complex II (succinate dehydrogenase) is the smallest mitochondrial ETC complex that links directly the ETC to the tricarboxylic acid (TCA) cycle. Unlike other respiratory complexes, Complex II is totally encoded by nuclear DNA and does not move protons to the IMM space (Bezawork-Geleta et al., 2017). This complex has a mass of 124 kDa and is composed of two hydrophilic subunits, three Fe-S centers, a flavoprotein, and two hydrophobic subunits, which contain one heme b and the binding site for Q (Sun et al., 2005). In the TCA cycle, the conversion of succinate to fumarate is coupled by the reduction of FAD to FADH_2_ in Complex II. FADH_2_ transfers two electrons to Fe–S clusters, which pass them to Q (Bezawork-Geleta et al., 2017). Despite many studies that investigated the effects of Cd on ETC Complex II (Liu and Liun, 1990; Fariss, 1991; Jay et al., 1991; Korotkov et al., 1998; Belyaeva et al., 2001, 2011; Casalino et al., 2002; Wang et al., 2004; Modi and Katyare, 2009), the mechanisms of Cd-induced Complex II injury are not fully elucidated. Many years ago, Toury et al. (1985) demonstrated a fall in Complex II activity in chronically treated rats exposed to Cd. In the same study, it was shown that isolated mitochondria from liver, kidney, and muscle of Cd-treated rats maintained partial energy coupling but displayed a rapid early fall in Complex IV activity followed by a partial restoration after 6 months of treatment and a progressively decreasing Complex II (Toury et al., 1985). Complex II inhibition blocks electron transfer and electron accumulation at Complex II, resulting in superoxide anion production. The key role of Complex II in ROS production was confirmed by Quinlan et al. (2012), showing that ROS from Complex II originates from the flavin site of the enzyme. Moreover, it has been demonstrated that a high succinate concentration associated with a high membrane potential-induced reverse electron transfer from Complex II into Complex I with superoxide production (Muller et al., 2008). Complex II inhibition by Cd was confirmed in mitochondria isolated from the liver, brain, and heart, even though only Complex III was demonstrated responsible for ROS production (Wang et al., 2004). It has been recently demonstrated that malonate (Complex II inhibitor) exerted protective effects against Cd-induced necrosis in rat hepatocellular carcinoma AS-30D cells and reduced ROS production induced by Cd in PC12 cells, suggesting the key role of Complex II in Cd-induced mitochondrial alterations (Belyaeva, 2018).

## Cadmium and Complex III

Complex III (coenzyme Q-cytochrome c reductase, also known as the cytochrome *bc*_1_ complex) mediates electron transport from Q to cyt c *via* a peculiar pathway known as the Q cycle. Complex III is a pear-shaped dimer, with 11 subunits per monomer. Cyt b, cyt c_1_, and 2Fe–2S cluster are the catalytical subunits. The cyt b contains two Q binding sites, Q_o_ near the intermembrane space and Q_i_ at the matrix side of the membrane (Wu et al., 2016). QH_2_ is oxidized to semi-ubiquinone (Q^•^^–^) after transferring an electron to the 2Fe–2S cluster; simultaneously, two protons are transported from the matrix across the IMM. Then, the 2Fe–2S cluster transfers the electron to cyt c *via* cyt c_1_. At the Q_o_ site, the formed Q^•^^–^ transfers the electron to another Q molecule *via* cyt b, forming Q^•^^–^ at the Q_i_ site. Finally, a second QH_2_ molecule is oxidized at the Q_o_ site, and two other protons are released across the IMM. This second QH_2_ molecule transfers one electron to the 2Fe–2S cluster and the other to Q^•^^–^ at Q_i_ site *via* cyt b producing QH_2_ (Crofts et al., 2017).

It has been shown that the toxic effect of Cd has been ascribable to mitochondrial ETC inhibition by impairing electron flow through Complex III (Miccadei and Floridi, 1993). Complex III has shown a high sensitivity to Cd that leads to around 77% inhibition and, as reported by electron spin resonance (ESR) experiments, Complex III might be the only site of ROS production induced by Cd (Wang et al., 2004). Cd may bind between Q^•^^–^ and cyt b of the Q_o_ site of Complex III, resulting in Q^•^^–^ accumulation at the Q_o_ site. Q^•^^–^ is very unstable and can transfer one electron to molecular oxygen forming superoxide (Wang et al., 2004). The protective effect against Cd-induced cytotoxicity of 2,6-dichloroindophenol (DCIP), a complex III bypass agent, confirms these data (Monroe and Halvorsen, 2009). Moreover, it has been suggested that ROS produced by Complex III were functionally linked to the mitochondrial PTP (Armstrong et al., 2004) and that Complex III and Complex I might be involved in the mitochondrial PTP promoted by Cd (Belyaeva et al., 2012).

## Cadmium and Complex IV

Complex IV, also called cyt c oxidase (COX), transfers electrons from cyt c to reduce oxygen. Simultaneously, Complex IV also drives four protons across the IMM. Mammalian Complex IV has a mass of 204 kDa and contains two hemes (a and a_3_) and three copper ions, two in the Cu_A_ center and one in the Cu_B_ site.

It has been shown in male Sprague-Dawley rats that Cd at low doses decreases the activity of Complex IV in liver mitochondria, but not in those of the kidney (Müller and Stacey, 1988). These data are confirmed, in mitochondria isolated from the liver of male Wistar rats, by the observation that Cd inhibited the ETC downstream Q (Ciapaite et al., 2009), supporting the results of Toury et al. (1985) that showed in mitochondria from the liver, kidney, and muscle of Cd-treated rats a significant decrease in cyt c oxidase activity. The reduced cyt c oxidase activity was also highlighted in the proximal tubular epithelial cells of rats subjected to prolonged Cd intoxication (Takaki et al., 2004). Moreover, in MDA-MB231 tumor cells, it was found that mRNA and protein levels of COXII and COXIV subunits decreased after long-term treatment with Cd, while short Cd exposure showed no effects (Cannino et al., 2008). More recently, Monteiro et al. (2018) demonstrated the Complex I and IV inhibition following Cd treatment in human MG-63 osteoblast cell line, confirming the key role of Cd in cyt c oxidase inhibition.

## Concluding Remarks

Cd is an environmental pollutant shown to display several undesirable effects on human health, targeting numerous organs and tissues. The molecular mechanisms responsible for most of the biological effects of Cd are not fully elucidated, and the toxicity targets have not been completely identified. Several studies focused their attention on mitochondria, which seem to represent the main intracellular targets of Cd. In particular, the different four complexes present in ETC have been suggested to play a key role in Cd-induced cytotoxicity, which is mainly displayed by mitochondrial ETC enzyme inhibition, enhanced ROS production, and uncoupling effect. However, other studies are needed to ascertain the dose- and exposure-dependent effects of Cd on *in vivo* experimental models.

## Author Contributions

JJVB, MB, and CF conceived the structure of the manuscript and drafted the manuscript. AP, MG, and NT critically revised the manuscript. All authors approved the final version of the manuscript.

## Conflict of Interest

The authors declare that the research was conducted in the absence of any commercial or financial relationships that could be construed as a potential conflict of interest.
